# Psychosexual distress following routine primary human papillomavirus testing: a longitudinal evaluation within the English Cervical Screening Programme

**DOI:** 10.1111/1471-0528.16460

**Published:** 2020-09-02

**Authors:** KF Bennett, J Waller, E McBride, AS Forster, G Di Gessa, H Kitchener, LAV Marlow

**Affiliations:** ^1^ Cancer Communication and Screening Group Department of Behavioural Science and Health University College London London UK; ^2^ Cancer Prevention Group School of Cancer and Pharmaceutical Sciences King's College London London UK; ^3^ Department of Epidemiology and Public Health University College London London UK; ^4^ Women's Cancer Centre Institute of Cancer Sciences University of Manchester Manchester UK

**Keywords:** Cervical screening, human papillomavirus, psychosexual distress

## Abstract

**Objective:**

To assess psychosexual distress over a 12‐month period among women receiving different human papillomavirus (HPV) and cytology results in the context of the English HPV primary screening pilot.

**Design:**

Longitudinal, between‐group study.

**Setting:**

Five sites in England where primary HPV testing was piloted.

**Population:**

Women aged 24–65 years (*n* = 1133) who had taken part in the NHS Cervical Screening Programme.

**Methods:**

Women were sent a postal questionnaire soon after receiving their screening results (baseline) and 6 and 12 months later. Data were analysed using linear regression models to compare psychosexual outcomes between groups receiving six possible combinations of HPV and cytology screening results, including a control group with normal cytology and no HPV test.

**Main outcome measures:**

Psychosexual distress, assessed using six items from the Psychosocial Effects of Abnormal Pap Smears Questionnaire (PEAPS‐Q).

**Results:**

At all time points, there was an association between screening result group and psychosexual distress (all *P* < 0.001). At baseline, mean psychosexual distress score (possible range: 1–5) was significantly higher among women with HPV and normal cytology (*B* = 1.15, 95% CI 0.96–1.34), HPV and abnormal cytology (*B* = 1.02, 95% CI: 0.78–1.27) and persistent HPV (*B* = 0.90, 95% CI 0.70–1.10) compared with the control group (all *P* < 0.001). At the 6 and 12 month follow ups the pattern of results were similar, but coefficients were smaller.

**Conclusions:**

Our findings suggest receiving an HPV‐positive result can cause psychosexual distress, particularly in the short‐term. Developing interventions to minimise the psychosexual burden of testing HPV‐positive will be essential to avoid unnecessary harm to the millions of women taking part in cervical screening.

**Tweetable abstract:**

Receiving an HPV‐positive result following primary HPV testing can cause psychosexual distress, particularly in the short‐term.

## Tweetable abstract

Receiving an HPV‐positive result following primary HPV testing can cause psychosexual distress, particularly in the short‐term.

## Introduction

Several countries, including England, have introduced primary human papillomavirus (HPV) testing for cervical screening because of its higher sensitivity for identifying high‐grade precancerous disease compared with cytology‐based testing.^1^, ^2^, ^3^ All women taking part in cervical screening in England are informed whether they are positive or negative for high‐risk HPV. When HPV is found, the residual sample is used for cytology triage. Women with HPV and normal cytology are re‐screened after 12 months and those with abnormal cytology are referred for colposcopy.^4^ Because HPV is sexually transmitted, primary HPV testing may have implications for psychosexual functioning.^5^

Psychosexual functioning includes feelings, worries and concerns that relate to, or impact on, sexual behaviour or sexual relationships. A systematic review of 25 studies^5^ identified a range of HPV‐related psychosexual concerns in the qualitative literature. These included concern about where the infection came from and transmitting HPV to a sexual partner. For some women, testing HPV‐positive had an impact on interpersonal and sexual relationships. However, quantitative studies found mixed evidence for differences in psychosexual outcomes between HPV‐positive women and comparison groups (usually those not tested for HPV or those with an HPV‐negative result).

Previous studies exploring psychosexual functioning following HPV testing have all been carried out in co‐testing contexts in England, and never in the context of HPV primary screening.^6^, ^7^, ^8^ One study found that HPV‐positive women were more likely to report feeling worse about their sexual relationships a week after receiving their result than HPV‐negative women, irrespective of their cytology result.^8^ Another compared three groups of women with abnormal cytology and different HPV results (HPV‐positive, HPV‐negative and no HPV test).^6^ Six months after receiving their test results, sexual worries were significantly higher among HPV‐positive women than in the other two groups. One longitudinal Taiwanese study of HPV‐positive women found that impact on sexual relationships appeared to decline between 1 and 6 months after screening but remained similar at 6 and 12 months.^9^

Evaluating psychosexual distress following receipt of different HPV and cytology results will help to establish whether receiving particular results causes concern or has an adverse effect on women's relationships. Understanding the time‐points at which the impact is greatest could inform decisions about the timing of interventions. The aim of this study was to assess psychosexual distress following primary HPV testing among women receiving different HPV and cytology results, at three time‐points over a year.

## Methods

### Study design and population

Data were collected as part of the Psychological Impact of Primary Screening for HPV (PIPS) study (details reported elsewhere^10^), which was funded by Public Health England (PHE). A between‐group design was used to assess women at three time‐points: shortly after receiving their screening result (‘baseline’), and 6 and 12 months later. Participants included screening eligible women (i.e. those aged 24–65 years) who had taken part in the NHS Cervical Screening Programme in one of five primary HPV screening pilot sites. Potential participants received invitation packs by post within 3weeks of receiving their screening result. Those who wished to take part returned a completed consent form and questionnaire booklet. Participants who returned a consent form were mailed questionnaire packs 6 and 12 months later. Patients were not involved in the development of this research.

Of the 5488 women who were invited to take part in the study, 21% (*n* = 1154) returned a questionnaire booklet at baseline. Participants returning a questionnaire >90 days after date of identification and those who were aged >65 years and ineligible to take part in the study were excluded (*n* = 21). Of the remaining 1133 participants, 1132 consented to receive follow‐up questionnaires, 67% (*n* = 768) returned a questionnaire booklet at 6 months and 47% (*n* = 542) at 12 months. Altogether, 40.3% (*n* = 456) returned questionnaire booklets at baseline, 6 months and 12 months. Women were included in the analyses if they returned a questionnaire at one or more time‐points. Please see Figure S1 for an overview of recruitment and response.

Three groups of women were recruited following their first HPV test: those who tested HPV‐negative, those who were HPV‐positive with normal cytology (HPV‐positive, normal cytology) and those who were HPV‐positive with abnormal cytology (HPV‐positive, abnormal cytology). In addition, two groups of women who had initially tested positive for HPV and were attending their 12‐month follow‐up appointment were recruited: those who were still found to have HPV (HPV persistent), and those who tested HPV‐negative at the follow‐up appointment (HPV cleared). A group of women who had taken part in cytology‐based screening and had received a normal result were recruited as a control group. Throughout this paper, when we refer to screening result, we mean one of the five groups we recruited based on combinations of HPV and cytology test results that women would receive in the screening programme.

### Measures

The primary outcome measures for the PIPS study (anxiety and distress) are reported elsewhere.^11^ Psychosexual functioning was a secondary outcome, assessed using six items, five of which were taken from the Psychosocial Effects of Abnormal Pap Smears Questionnaire (PEAPS‐Q), a validated measure of distress experienced by women undergoing follow‐up investigation after an abnormal Pap smear result.^12^ These items measured two dimensions of psychosexual distress: worry about infectivity (2 items) and effect on sexual relationships (3 items). An additional item asked: ‘Have you been worried about whether your test result would have a bad effect on your relationship with your partner?’ This item was taken from Maissi et al.^6^ All six items used a 5‐point Likert response scale: Not at all (1), A little (2), A fair bit (3), Quite a lot (4), Very much (5), with an additional ‘not applicable’ option.

Overall psychosexual distress was calculated as the mean of all six items, as they showed good internal reliability (α = 0.93, *n* = 898). The potential range was 1–5, with higher scores indicating greater distress. Only women who had responded to all six psychosexual items were included in these analyses: 79% (*n* = 898) at baseline, 76% at 6 months (*n* = 581) and 78% at 12 months (*n* = 418). As the aim of the study was to assess the prevalence and magnitude of psychosexual distress following HPV testing, we excluded women who answered ‘not applicable’ to one or more questions (19% [*n* = 214] at baseline, 22% [*n* = 167] at 6 months and 21% [*n* = 113] at 12 months).

Socio‐demographic variables including self‐reported ethnicity (white, ethnic minority, prefer not to say), educational attainment (degree or higher, qualification below degree, no formal qualifications) and relationship status (current partner versus no partner) were collected. Age and Index of Multiple Deprivation (IMD) quintile (a postcode‐based measure of relative deprivation for small areas in England^13^) were collected from NHS clinical records.^10^ Socio‐demographic variables were collected at baseline only.

### Analyses

Analyses were carried out using SPSS v22 (IBM Corp, Armonk, NY, USA) and STATA SE v15 (StataCorp LLC, College Station, TX, USA).

We used linear regression models to explore the association between screening result group and psychosexual distress cross‐sectionally at baseline, 6 and 12 months.

We also used conditional change linear regression models to examine changes in psychosexual distress by screening result group between baseline and 6 and 12 months. Using this approach, the baseline psychosexual distress score is controlled for, so the regression coefficients indicate how the screening result group is associated with changes in psychosexual distress over time.^14^

In all models, we adjusted for baseline demographic characteristics (age, ethnicity, education, marital status and IMD quintile) and applied weights to adjust for the possibility that the approached sample may not have been representative of the screening population in the HPV testing pilot sites (details described elsewhere).^11^

We also explored between‐group differences for each individual PEAPS‐Q item at baseline, 6 and 12 months. All women who had responded to an item, regardless of whether they were excluded from the overall psychosexual distress analyses, were included in the individual item analyses. In the original PEAPS‐Q development paper, Bennetts et al.^12^ classified a woman as ‘distressed’ if she responded ‘Quite a lot’ or ‘Very much’ to an item. We dichotomised responses in this way, coding women as ‘distressed’ (if they responded 4 or 5) or ‘not distressed’ (if they responded 1–3). The percentage of women reporting 'psychosexual distress’ was calculated for each item and is reported by screening result group. Where we report ‘distress’ we are referring to psychosexual distress rather than general psychological distress.

## Results

Characteristics of the 1088 women who responded to at least one psychosexual item at baseline are shown in Table 1. Demographic characteristics of the sample by screening result group are presented elsewhere.^11^At baseline, women had a mean age of 41 years, were predominantly white (92%), half had a qualification below degree level (49%) and most had a current partner (79%).

**Table 1:** Demographic characteristics of the sample included in analysis at baseline (*n* = 1088)*, 6‐month follow up (*n* = 734) and 12‐month follow up (*n* = 503)

	Baseline	6 mo	12 mo
	*n* (%)	*n* (%)	*n* (%)
**Screening result group**			
HPV‐negative	233 (21.4)	176 (24.0)	115 (22.9)
HPV‐positive, normal cytology	251 (23.1)	169 (23.0)	105 (20.9)
HPV‐positive, abnormal cytology	167 (15.3)	106 (14.4)	70 (13.9)
HPV persistent	177 (16.3)	115 (15.7)	88 (17.5)
HPV cleared	63 (5.8)	41 (5.6)	34 (6.8)
Control (normal cytology)	197 (18.1)	127 (17.3)	91 (18.1)
Age (mean years/SD)	40.84 (SD = 11.68)	42.78 (SD = 11.70)	42.70 (SD = 11.86)
**Ethnicity**			
White (British or other)	982 (92.0)	676 (92.1)	464 (92.2)
Ethnic minority	83 (7.8)	43 (5.9)	32 (6.4)
Prefer not to say	2 (0.2)	0 (0)	0 (0)
**Education**			
Degree or higher	470 (44.3)	329 (44.8)	231 (45.9)
Qualification below degree	516 (48.6)	344 (46.9)	227 (45.1)
No formal qualifications**	75 (7.1)	46 (6.3)	36 (7.2)
**Marital status** ***			
Current partner	841 (78.7)	566 (77.1)	394 (78.3)
No partner	228 (21.3)	155 (21.1)	102 (20.3)
**IMD quintile**			
1 (most deprived)	165 (16.4)	92 (12.5)	62 (12.3)
2	204 (20.2)	126 (17.2)	85 (16.9)
3	265 (26.3)	185 (25.1)	149 (29.6)
4	182 (18.1)	135 (18.4)	95 (18.9)
5 (least deprived)	192 (19.0)	139 (18.9)	83 (16.5)

* The samples included in these analyses differ from the total sample at each time‐point, as only women responding to one or more of the psychosexual items are included.

** No formal qualifications included those with no qualifications and those still studying.

*** Marital status: ‘current partner’ included those who were married, in a civil partnership, living with a partner or in a relationship. ‘No partner’ included those who were single, divorced, separated or widowed.

### Psychosexual distress across result groups

**Figure 1:**
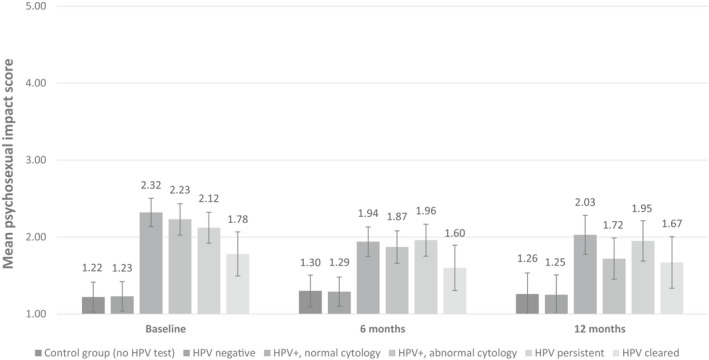
Adjusted* mean scores for psychosexual distress at baseline, 6 mo and 12 mo by result group with 95% confidence intervals (unweighted). *Adjusted for age, ethnicity, marital status, education and IMD.

Adjusted and weighted beta coefficients (with 95% confidence intervals) and *P*‐values for the relation between psychosexual distress and result group cross‐sectionally at baseline, 6 and 12 months are presented in Table 2. Adjusted mean psychosexual distress scores for each group at baseline, 6 and 12 months are presented in Figure 1.

**Table 2:** Cross‐sectional associations between psychosexual distress and screening result group and change in psychosexual distress (weighted^1^ and adjusted^2^ ^,^ ^3^)

	Cross‐sectional associations^2^			Change in psychosexual distress^3^	
	At baseline	At 6 mo	At 12 mo	By 6 mo	By 12 mo
	*B*^4^ (95% CI)	*B*^4^ (95% CI)	*B*^4^ (95% CI)	*B*^4^ (95% CI)	*B*^4^ (95% CI)
**Screening result group**					
Control group (normal cytology)	Reference	Reference	Reference	Reference	Reference
HPV‐negative	0.001 (−0.090,0.087)	−0.016 (−0.125,0.092)	0.004 (−0.086,0.094)	0.022 (−0.118,0.161)	0.091 (−0.267, 0.209)
HPV‐positive, normal cytology	1.148 (0.960,1.336)***	0.675 (0.493,0.857)***	0.810 (0.558,1.061)***	−0.450 (−0.636,−0.263)***	−0.543 (−0.776,−0.310)***
HPV‐positive, abnormal cytology	1.014 (0.771,1.256)***	0.639 (0.374,0.903)***	0.503 (0.217,0.788)**	−0.438 (−0.700,−0.176)**	−0.325 (−0.607,−0.044)*
HPV persistent	0.905 (0.705,1.105)***	0.676 (0.434,0.918)***	0.690 (0.471,0.909)***	−0.471 (−0.694,−0.250)***	−0.463 (−0.676,−0.250)***
HPV cleared	0.616 (0.330,0.901)***	0.239 (−0.026,0.504)	0.368 (0.049,0.686)*	−0.108 (−0.364,0.147)	−0.174 (−0.457,0.109)
Constant	0.866 (0.584,1.149)***	0.829 (0.530,1.128)***	0.766 (0.417,1.115)***	−0.465 (−0.782,−0.147)**	−0.538 (−0.892,−0.184)**
Model *F*	22.90	9.89	7.35	21.34	20.66
Number of observations	801	520	383	517	382
*R* ^2^	0.281	0.222	0.221	0.626	0.647

^1^ Weighted by age group and IMD quintile.

^2^ Cross‐sectional models were adjusted for age, ethnicity, marital status, education and IMD.

^3^ Conditional change models were adjusted for age, ethnicity, marital status, education, IMD and baseline psychosexual distress score.

^4^ Beta coefficients (with 95% CI) indicating the degree of change in psychosexual distress for each screening result group compared to the reference group (i.e. the control group).

* *P* < 0.05.

** *P* < 0.01.

*** *P* < 0.001.

At baseline, there was a significant association between screening result and psychosexual distress (*P* < 0.001). Compared with the control group, psychosexual distress was higher among women in the HPV‐positive, normal cytology group (by 1.15 points), the HPV‐positive, abnormal cytology group (by 1.01 points), the HPV persistent group (by 0.91 points) and the HPV cleared group (0.62 points higher; all *P* < 0.001). There was no significant difference between the control group and the HPV‐negative group (*P* = 0.974) (see Table 2).

At the 6 and 12 month follow up, the association between result group and psychosexual distress remained significant (*P* < 0.001). The pattern of results was similar to that seen at baseline, although coefficients were smaller. Psychosexual distress remained highest and significantly different from the control group (*P* < 0.001) in all three HPV‐positive groups. Compared with the control group, psychosexual distress was higher among women in the HPV‐positive, normal cytology group (by 0.68 points at 6 months and 0.81 points at 12 months), the HPV‐positive, abnormal cytology group (by 0.64 points at 6 months and 0.50 points at 12 months) and the HPV persistent group (by 0.68 points at 6 months and 0.69 points at 12 months). For the HPV cleared group, psychosexual distress was not significantly higher than the control group at 6 months (*P* = 0.076) but was at 12 months (by 0.37 points, *P* = 0.024). There was no significant difference between the control group and the HPV‐negative group at 6 months (*P* = 0.767) or 12 months (*P* = 0.931).

Adjusted and weighted beta coefficients (with 95% confidence intervals) and *P*‐values for the association between changes in psychosexual distress by screening result group at 6 and 12 months are presented in Table 2. There were significant reductions in psychosexual distress among women in the HPV‐positive, normal cytology group (by 0.45 points at 6 months and 0.54 points at 12 months), the HPV‐positive, abnormal cytology group (by 0.44 points at 6 months and 0.33 points at 12 months) and the HPV persistent group (by 0.47 points at 6 months and 0.46 points at 12 months). There were no significant changes in psychosexual distress among women in HPV cleared group at 6 months (*P* = 0.405) or 12 months (*P* = 0.227) or the HPV‐negative group at 6 months (*P* = 0.767) or 12 months (*P* = 0.931).

### Psychosexual distress by individual item

The overall percentage of participants who were categorised as ‘distressed’ for each item at baseline, 6 and 12 months is presented in Table 3. The table also shows the proportion who were distressed at baseline by screening result group (see Table S1 and S2 for 6 and 12 month follow‐up data by group).

**Table 3:** Percentage ‘distressed’* for individual psychosexual questions at baseline, 6 mo and 12 mo and by screening result group at baseline

Have you been worried…	% (*n*) ‘distressed’								
	All women testing HPV‐positive**			HPV‐positive, normal cytology	HPV‐positive, abnormal cytology	HPV persistent	Control group	HPV‐negative	HPV cleared
	Baseline	6 mo	12 mo	Baseline					
	*n* = 1088	*n* = 734	*n* = 503	*n* = 251	*n* = 167	*n* = 177	*n* = 197	*n* = 233	*n* = 63
…whether you should continue having sex?	16.8 (93)	7.2 (26)	9.7 (24)	18.8 (44)	18.8 (29)	12.0 (20)	0 (0)	0.4 (1)	6.6 (4)
…others think you have had more sexual partners than you should?	16.8 (96)	10.5 (39)	11.2 (29)	17.9 (42)	16.1 (26)	15.9 (28)	0 (0)	1.4 (3)	9.7 (6)
…about whether your test result would have a bad effect on your relationship with your partner?	18.7 (98)	10.5 (36)	9.4 (22)	20.5 (45)	16.2 (23)	18.6 (30)	1.1 (2)	0.5 (1)	12.3 (7)
…whether having sex will make the problem worse?	14.7 (82)	10.0 (37)	7.2 (18)	16.5 (38)	15.2 (24)	11.8 (20)	1.1 (2)	1.0 (2)	9.8 (6)
… that you could give the problem to a sexual partner?	27.6 (154)	18.1 (66)	16.6 (42)	28.3 (66)	26.3 (41)	27.8 (47)	2.3 (4)	0.5 (1)	16.4 (10)
…a sexual partner will think they can catch the problem from you?	27.2 (150)	16.5 (60)	16.6 (41)	31.0 (72)	24.3 (37)	24.6 (41)	2.9 (5)	0 (0)	15.0 (9)

* Percentage of women who responded ‘Quite a lot’ or ‘Very much’ on the Likert scale.

** Includes women in the HPV‐positive, normal cytology, HPV‐positive, abnormal cytology and HPV persistent groups.

At baseline, the percentage who were distressed was lowest among the control group (range: 0–2.9%) and the HPV‐negative group (range: 0–1.4%) and highest among the three HPV‐positive groups (HPV‐positive, normal cytology range: 16.5–31%; HPV‐positive, abnormal cytology range: 15.2–26.3%; HPV persistent range: 11.7–27.9%). Overall, the percentage classed as distressed decreased over time for all items. At all three time‐points, distress was most prevalent for the two items assessing worry about infectivity.

## Discussion

### Main findings

Women testing positive for HPV at cervical screening reported higher psychosexual distress than those receiving a normal cytology result with no HPV test. The differences were observed immediately after screening and were attenuated but remained significant 6 and 12 months later. HPV‐negative women who had tested positive 12 months previously (‘HPV cleared’) also had higher psychosexual distress immediately after their HPV‐negative result and 12 months later. Our findings suggest that psychosexual distress declines over time among HPV‐positive women in the first 6 months.

### Strengths and limitations

This is the first longitudinal study to explore psychosexual distress following routine primary HPV screening among women with different HPV and cytology results. It is also the first study to include a group of women who had previously tested HPV‐positive and were found to have cleared the infection 12 months later. The main limitation of the study was the low response rate, which ranged by screening result group from 16% in those not tested for HPV to 28% in those with persistent HPV. In addition, a third of women who participated at baseline did not complete the 6‐month follow up, and a further 20% did not complete the 12‐month follow up. We have no psychosexual functioning data for the women who did not respond, so we cannot rule out the possibility that response to the survey was systematically associated with psychosexual distress. However, we were able to weight our data to the screening population in the HPV testing pilot sites for age and IMD, helping to improve representativeness with respect to demographic characteristics.

This study consisted predominantly of women of white ethnicity, which reflects the screening population in the UK. Previous research suggests that the stigma of testing HPV‐positive may be greater among some minority ethnic groups.^15^, ^16^ Research specifically designed to explore psychosexual distress following HPV testing in minority ethnic groups is needed.

### Interpretation

Our study was conducted in the context of the English HPV primary screening pilot. Although carried out in a different setting, our findings are similar to those by Hsu et al.,^9^ who found that the impact on sexual relationships declined between 1 and 6 months and remained similar between 6 and 12 months. They are also consistent with Maissi et al.,^6^ who found that 6 months after receiving results, psychosexual outcomes were virtually the same for women testing HPV‐negative and those not tested for HPV, but significantly higher for women who were HPV‐positive. Psychosexual distress scores for HPV‐positive women in our study were slightly lower than in Maissi et al.;^6^ however, increased awareness and knowledge of HPV since 2005 may have helped to reduce the negative psychosexual consequences of testing HPV‐positive.

The percentage of women classified as distressed for each individual item at baseline ranged from 9 to 17%. Distress was more prevalent than reported by Bennetts et al.,^12^ who classified 3–11% of women as distressed during follow‐up investigation after an abnormal Pap smear result. The diagnosis of a sexually transmitted infection can be associated with feelings of stigma and shame^17^, ^18^, ^19^ and it is possible that the stigma of having HPV may have a greater impact on psychosexual functioning than receiving an abnormal cytology result does. This is supported by qualitative research which suggested some women chose not to disclose their HPV infection to their partner and instead focused on their abnormal cytology result, which did not carry the same negative connotations.^15^

The most commonly endorsed items at all three time‐points concerned infectivity, with around 25% of women who were HPV‐positive indicating infectivity concerns at baseline. This finding is consistent with a synthesis of qualitative research exploring the psychosexual impact of testing HPV‐positive.^5^ Transmission and the impact of HPV on a sexual partner have been identified as key topics that women want more information on, and uncertainty about these aspects of HPV can influence women's psychological response to HPV.^20^ This highlights the importance of ensuring that common questions and concerns about infectivity and transmission are addressed in materials for women who test HPV‐positive.

At baseline, psychosexual distress was highest among women in the HPV‐positive with normal cytology group. Testing HPV‐positive with normal cytology is a new result created by the primary HPV screening pathway, and because knowledge of HPV can be low^21^ it is possible that women unfamiliar with this new result lack understanding about what it means for their sexual relationships. In addition, with no abnormal cytology result, there may be greater focus on HPV which, as a sexually transmitted infection (STI), may have greater potential for psychosexual impact. Psychosexual distress may also be exacerbated by the prospect of having to wait a year to see whether the infection has cleared. Reassuringly, psychosexual distress declined between baseline and 6 months in this group.

At 12 months, psychosexual distress was still highest among women in the HPV‐positive with normal cytology group. However, there were smaller reductions in psychosexual distress between baseline and 12 months in the HPV‐positive with abnormal cytology group than in the HPV‐positive with normal cytology group. It is possible that women in the HPV‐positive with normal cytology group who returned the 12‐month questionnaire were the most concerned (responder bias), which is why, cross‐sectionally, psychosexual distress was highest in this group.

Compared with women not tested for HPV, the HPV cleared group had significantly higher psychosexual distress at baseline and this remained significantly higher 12 months later. Although the mean psychosexual distress score was not as high in the HPV cleared group as the three HPV‐positive groups, this suggests that women who had previously tested HPV‐positive may still have residual psychosexual concerns, despite an HPV‐negative result. A qualitative study^22^ exploring women's experiences of repeat HPV testing found that some had concerns about the infection recurring and worried that it was lying dormant and might reappear in the future. Future research should explore psychosexual concerns specific to this group.

Our findings suggest that receiving an HPV‐positive result can lead to elevated psychosexual distress, particularly in the short‐term. It should be noted that the differences between the three HPV‐positive groups and the control group were small at baseline (a difference of ~1 point on a 5‐point scale) and smaller still at follow up (<1 point difference).For most women, it is unlikely that testing HPV‐positive would have a meaningful impact on psychosexual functioning. There is no established ‘normal’ range for the PEAPS‐Q, so it is difficult to determine whether these differences are clinically significant. Although we are unable to determine the number of women who are likely to present with psychosexual concerns requiring clinical services (e.g. psychosexual counselling), the study suggests that there are women who have concerns therefore efforts to address these at a population level are important. As the individual psychosexual items suggest concerns about infectivity are relatively common, simple interventions such as including information about this in results letters and leaflets for women who test HPV‐positive should be considered.

It is possible that women may have additional psychosexual concerns not captured by the items we used. Future research should use qualitative methodology to explore the full range of psychosexual questions and concerns among women taking part in HPV‐based cervical screening. This additional insight may help to ensure that screening information materials and results letters meet the needs of women with different HPV and cytology results.

## Conclusion

This study suggests that testing HPV‐positive can result in elevated psychosexual distress, particularly in the short‐term. It is reassuring that psychosexual distress decreased over time; however, even at the 12‐month follow up there were small differences between the control group (who were not tested for HPV) and women who were HPV‐positive or had cleared a previous HPV infection. Developing interventions to minimise the psychosexual burden of testing positive for HPV will be essential to avoiding unnecessary harm to the millions of women taking part in cervical screening.

### Disclosure of interests

KB received grants from the Medical Research Council (MRC) during the conduct of the study. JW, AF and LM received grants from Cancer Research UK during the conduct of the study. EM received grants from the National Institute for Health Research (NIHR) during the conduct of the study. Public Health England (PHE) commissioned the project and paid salary costs for EM during the data collection period as well as all research costs. GDG and HK have nothing to disclose. Completed disclosure of interest forms are available to view online as supporting information.

### Contribution to authorship

JW, AF, HK and LM conceived the study. JW, EM, AF and LM developed the measures. KB and GDG conducted the analyses. KB drafted the paper. All authors contributed to the final version of the manuscript.

### Details of ethics approval

Health Research Authority approval was obtained on 26 September 2016 and approval from London‐Surrey NHS Research Ethics Committee (REC) on 30 August 2016 (Research Ethics Committee reference: 16/LO/0902). Section 251 approval was also obtained from the Confidentiality Advisory Group (CAG) for use of patient name and address without consent for the purposes of participant approach on 24 August 2016 (Confidentiality Advisory Group reference: 16/CAG/0047).

### Funding

The study was funded by Public Health England (PHE). KB is funded by a Medical Research Council Studentship (MR/N013867/1). JW and LM are funded by Cancer Research UK (C7492/A17219). AF is also funded by Cancer Research UK (C49896/A17429). EM was funded by PHE from 3 January 2016 to 30 September 2017. EM was funded by the National Institute of Health Research (NIHR) from 1 October 2017 (DRF‐2017‐10‐105). The views expressed in this paper are not necessarily those of the NHS or the Department of Health.

### Acknowledgements

We would like to thank the NHS clinical laboratory managers and staff at the HPV primary screening pilot sites who helped us gain HRA approvals and recruit participants (Kay Ellis, Christopher Evans, Nicola Fagan, Viki Frew, Miles Holbrook, Janet Parker, David Smith and Carol Taylor). We thank Ruth Stubbs and Karen Denton at Public Health England for facilitating the public facing aspects of the study and helping with HRA approvals. Thank you to Matejka Rebolj and Christopher Matthews at King’s College London who provided us with population‐level screening data to allow us to weight our sample, as well as providing helpful guidance at several points in the study. Thanks to Lauren Rockliffe who helped with participant recruitment and data entry. Finally, thank you to the women who kindly gave up their time to participate.

## Supporting information

**Figure S1.** An overview of recruitment and response

**Table S1.** Percentage ‘distressed’¹ for individual psychosexual questions by screening result group at 6‐month follow up

**Table S2.** Percentage ‘distressed’¹ for individual psychosexual questions by screening result group at 12‐month follow‐up

Supplementary Material

Supplementary Material

Supplementary Material

Supplementary Material

Supplementary Material

Supplementary Material

Supplementary Material
