# Supplementary material for: Psychosexual distress following routine primary human papillomavirus testing: a longitudinal evaluation within the English Cervical Screening Programme
Source: BJOG. 2020 Sep 2;128(4):745–54. doi: 10.1111/1471-0528.16460 (PMC8432156; doi:10.1111/1471-0528.16460)
Supplement: Supplementary file 1 — Figure S1. An overview of recruitment and response [file BJO-128-745-s007.pdf]

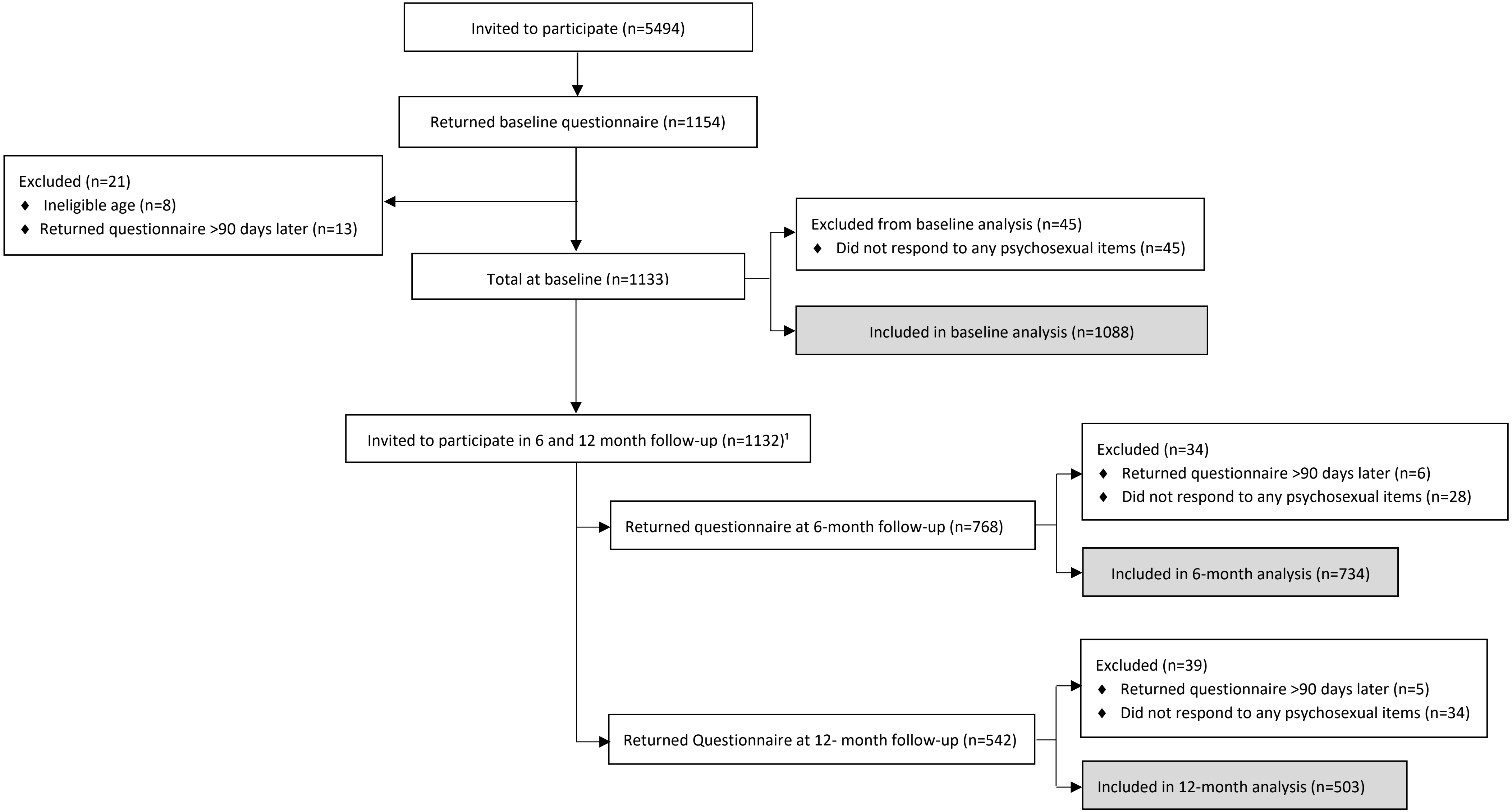

[1]One participant did not provide consent to be followed-up so was not invited to participate in the 6 and 12-month follow-ups.

Figure S1. An overview of recruitment and response
