# Supplementary material for: Psychosexual distress following routine primary human papillomavirus testing: a longitudinal evaluation within the English Cervical Screening Programme
Source: BJOG. 2020 Sep 2;128(4):745–54. doi: 10.1111/1471-0528.16460 (PMC8432156; doi:10.1111/1471-0528.16460)
Supplement: Supplementary file 2 — Table S1. Percentage ‘distressed’¹ for individual psychosexual questions by screening result group at 6‐month follow up [file BJO-128-745-s004.pdf]

Table S1. Percentage 'distressed'[1] for individual psychosexual questions by screening result group at 6-month follow-up

	% (n) 'distressed'						
	**Whole sample**	**Control group**	**HPV negative**	**HPV positive, normal cytology**	**HPV positive, abnormal cytology**	**HPV persistent**	**HPV cleared**
	**n=734**	**n=127**	**n=176**	**n=169**	**n=106**	**n=115**	**n=41**
Have you been worried…							
…whether you should continue having sex?	4.5 (33)	2.4 (3)	0.6 (1)	5.1 (8)	7.1 (7)	10.5 (11)	7.5 (3)
…others think you have had more sexual partners than you should?	5.4 (40)	0 (0)	0 (0)	4.9 (8)	17.8 (18)	12.1 (13)	2.6 (1)
…about whether your test result would have a bad effect on your relationship with your partner?	5.6 (41)	1.7 (2)	0.6 (1)	9.4 (14)	9.0 (8)	13.3 (14)	5.6 (2)
…whether having sex will make the problem worse?	6.1 (45)	2.7 (3)	0.7 (1)	7.0 (11)	13.0 (13)	11.7 (13)	10.5 (4)
… that you could give the problem to a sexual partner?	9.7 (71)	0.9 (1)	0.7 (1)	16.5 (26)	20.8 (20)	18.3 (20)	7.7 (3)
…a sexual partner will think they can catch the problem from you?	8.6 (63)	0 (0)	0.7 (1)	15.3 (24)	16.7 (16)	18.5 (20)	5.1 (2)

[1] Percentage of women who responded 'Quite a lot' or 'Very much' on the Likert scale
